# Association between serum insulin levels and heart failure-related parameters in patients with type 2 diabetes and heart failure treated with canagliflozin: a post-hoc analysis of the randomized CANDLE trial

**DOI:** 10.1186/s12933-022-01589-3

**Published:** 2022-08-08

**Authors:** Atsushi Tanaka, Takumi Imai, Michio Shimabukuro, Isao Taguchi, Akira Sezai, Shigeru Toyoda, Hirotaka Watada, Junya Ako, Koichi Node, the CANDLE trial investigators

**Affiliations:** 1 Department of Cardiovascular Medicine, Saga University, 5-1-1 Nabeshima, Saga, 849-8501 Japan; 2 Department of Medical Statistics, Graduate School of Medicine, Osaka Metropolitan University, Osaka, Japan; 3 Department of Diabetes, Endocrinology, and Metabolism, Fukushima Medical University, Fukushima, Japan; 4 Department of Cardiology, Dokkyo Medical University Saitama Medical Center, Koshigaya, Japan; 5 Department of Cardiovascular Surgery, Nihon University School of Medicine, Tokyo, Japan; 6 Department of Cardiovascular Medicine, Dokkyo Medical University School of Medicine, Mibu, Japan; 7 Department of Metabolism & Endocrinology, Juntendo University Graduate School of Medicine, Tokyo, Japan; 8 Department of Cardiovascular Medicine, Kitasato University School of Medicine, Sagamihara, Japan

**Keywords:** Type 2 diabetes, Chronic heart failure, Canagliflozin, Glimepiride, Insulin

## Abstract

**Background:**

Insulin resistance and hyperinsulinemia in patients with type 2 diabetes (T2D) are adversely associated with the development and worsening of heart failure (HF). Herein, we sought to investigate the effect of canagliflozin on insulin concentrations and the associations of changes in insulin concentrations with HF-related clinical parameters in patients with T2D and HF.

**Methods:**

This was a post-hoc analysis of the investigator-initiated, multicenter, open-label, randomized, controlled CANDLE trial for patients with T2D and chronic HF (UMIN000017669). The endpoints were the effects of 24 weeks of canagliflozin treatment, relative to glimepiride treatment, on insulin concentrations and the relationship between changes in insulin concentrations and clinical parameters of interest, including New York Heart Association (NYHA) classification. The effects of canagliflozin on those parameters were also analyzed by baseline insulin level.

**Results:**

Among the participants in the CANDLE trial, a total of 129 patients (canagliflozin, n = 64; glimepiride, n = 65) who were non-insulin users with available serum insulin data both at baseline and week 24 were included in this analysis. Overall, the mean age was 69.0 ± 9.4 years; 75% were male; the mean HbA1c was 6.8 ± 0.7%; and the mean left ventricular ejection fraction was 59.0 ± 14.1%, with parameters roughly balanced between treatment groups. Canagliflozin treatment significantly reduced insulin concentrations at week 24 (p < 0.001), and the between-group difference (canagliflozin minus glimepiride) in those changes was − 3.52 mU/L (95% confidence interval, − 4.85 to − 2.19; p < 0.001). Decreases in insulin concentrations, irrespective of baseline insulin level, were significantly associated with improvement in NYHA class in patients treated with canagliflozin.

**Conclusion:**

Our findings suggest that canagliflozin treatment in patients with T2D and HF ameliorated excess insulin overload, contributing to the improvement of clinical HF status.

*Trial registration:* University Medical Information Network Clinical Trial Registry, number 000017669, Registered on May 25, 2015.

**Supplementary Information:**

The online version contains supplementary material available at 10.1186/s12933-022-01589-3.

## Introduction

Insulin resistance and hyperinsulinemia play a central role in the pathogenesis of obesity and metabolic syndrome, including type 2 diabetes (**T2D**), resulting in an increased risk of cardiovascular disease (**CVD**) [1–3]. Such insulin abnormalities can also adversely affect cardiac function and serve as independent risk factors for incident heart failure (**HF**) [4, 5]. Conversely, excess inflammation in the visceral adipose tissue evoked in HF induces systemic insulin resistance, and the resulting hyperinsulinemia exacerbates HF by continuous activation of insulin signaling in cardiac tissue [6, 7]. Thus, insulin abnormalities and HF form a vicious cycle, and accordingly represent strong candidate targets of HF therapy.

Sodium-glucose cotransporter 2 (**SGLT2**) inhibitors have a unique mode of action to lower plasma glucose levels in an insulin-independent manner via an increase in urinary glucose excretion, which in turn can mitigate glucotoxicity and hyperinsulinemia [8]. Several experimental studies have also shown that SGLT2 inhibition attenuates systemic insulin resistance through complex actions in adipose tissues, liver, and skeletal muscles [9–12]. Clinical studies have demonstrated that SGLT2 inhibitor treatment induces favorable metabolic responses and improves insulin sensitivity in several tissues in patients with T2D [13–16].

Recent meta-analyses of cardiovascular (and/or renal) outcome trials (**CVOT**) with SGLT2 inhibitors showed that these agents reduce the risk of worsening HF and cardiovascular death in patients with T2D at high risk of cardiovascular events [17, 18]. More recent CVOTs also demonstrated that SGLT2 inhibitor therapy reduces the risk of HF-related clinical events specifically in patients with HF, irrespective of diabetes status [19, 20]. Given the close link between impaired insulin actions and HF, the correction of insulin resistance and hyperinsulinemia induced by SGLT2 inhibition likely contributed to the clinical benefits observed in those CVOTs [21]. However, the effects of SGLT2 inhibition on serum insulin concentrations and its relationship of SGLT2 inhibition with clinical impact remain poorly elucidated in those CVOTs, even in patients complicated with HF. Therefore, herein, we sought to investigate the effect of the SGLT2 inhibitor canagliflozin on serum insulin concentrations and the associations of such changes with HF-related clinical parameters of interest, using data obtained from the randomized CANDLE trial for patients with T2D and HF [22].

## Methods

### Study design and subjects

This was a post-hoc analysis of the CANDLE trial (UMIN000017669), an investigator-initiated, multicenter, prospective, randomized, open-label clinical trial in which the primary endpoint was the effect of 24 weeks of canagliflozin therapy, relative to glimepiride therapy, on N-terminal pro-brain natriuretic peptide **(NT-proBNP**) concentrations in patients with T2D and chronic HF (**CHF**) [22]. The CANDLE trial was approved by the institutional review boards of the individual sites and conducted in accordance with the Declaration of Helsinki. All participants provided written, informed consent prior to screening and randomization.

Details of the study design and inclusion and exclusion criteria have been reported previously [23]. Briefly, the key eligibility criteria were (i) adults, (ii) T2D, and (iii) CHF with New York Heart Association (**NYHA**) class I to III, with no change in NYHA class and background therapies for HF within 4 weeks prior to screening. Key exclusion criteria included type 1 diabetes, severe hepatic and/or renal dysfunction (estimated glomerular filtration rate [**eGFR**] < 45 mL/min/1.73m^2^ or on dialysis), NYHA class IV, history of diabetic ketoacidosis, diabetic coma, or hypoglycemic attack within 6 months prior to study enrollment, and history of CVD within 3 months prior to eligibility assessment.

Eligible participants were randomly allocated to receive either canagliflozin (100 mg daily) or glimepiride (starting dose 0.5 mg daily) add-on therapy at a ratio of 1:1 using a web-based minimization method balanced for age (< 65, ≥ 65 years), hemoglobin A1c (HbA1c) level (< 6.5%, ≥ 6.5%), and left ventricular ejection fraction (**LVEF**; < 40%, ≥ 40%) at the time of screening. All participants received the study therapy for 24 weeks. In participants assigned to the glimepiride group, adjustment of the glimepiride dose was allowed according to individual glycemic management and the local investigator’s judgment. Background medications for T2D, CHF, and other comorbidities were, in principle, unchanged during the study interval within clinically permissible ranges.

### Measurements and endpoints

The details of the original outcome measures in the CANDLE trial have been described previously [23]. Briefly, vital sign recording and blood sample collection were mandatory at baseline and at week 24. Routine laboratory data, including glycemic parameters were, in principle, collected in the early morning when fasting and measured at each local site. Based on a previous report showing that fasting levels of plasma insulin were 11.2 ± 6.0 mU/L in Japanese patients with T2D [24], serum insulin values > 20 mU/L were considered inappropriate for fasting conditions and thereby excluded from the present analysis. The homeostasis model assessment of insulin resistance (**HOMA-IR**) was calculated as serum insulin (mU/L) × plasma glucose (mg/dL)/405. NT-proBNP concentrations were measured at baseline and week 24 in a blinded manner at central commercial laboratories (SRL Inc., Tokyo, Japan). The percentage change in estimated plasma volume (**ePV**) from baseline to week 24 was calculate with the Strauss formula [25–27].

In the present analysis, we compared the effects of 24 weeks of canagliflozin therapy, relative to glimepiride, on serum insulin levels and other glycemic parameters. In addition, we assessed the relationship between changes in the insulin levels and other clinical parameters of interest, including NT-proBNP, obtained in the CANDLE trial. Furthermore, we compared the effects of two study therapies on those parameters by baseline serum insulin level. Among the prespecified full analysis set (**FAS**) of the CANDLE trial dataset, participants who were non-insulin users and had available serum insulin data (≤ 20 mU/L) both at baseline and week 24 were included in the present analysis.

### Statistical analysis

Baseline demographics and clinical characteristics are expressed as number (%) for categorical variables and as mean ± standard deviation or median [interquartile range] for continuous variables where appropriate. Comparisons between the treatment groups were made using linear regression models for changes in serum insulin levels and glycemic parameters from baseline to week 24. Associations between changes from baseline to week 24 in serum insulin concentrations and clinical parameters of interest were assessed by calculating Pearson’s correlation coefficients. To investigate the influence of baseline serum insulin levels on treatment effects for those parameters at week 24, data were analyzed using linear regression models for NT-proBNP and linear mixed models for other parameters in subgroups according to baseline serum insulin level. The ratio (canagliflozin vs. glimepiride) of the proportional change from baseline to week 24 in NT-proBNP was estimated based on a natural logarithmic scale [28]. A p*-*value for the interaction between the study treatments and baseline serum insulin category on the NYHA class was calculated using an ordinal logistic regression model analysis.

All statistical analyses were performed using R software, version 3.6.3 (R Foundation for Statistical Computing, Vienna, Austria) at a two-sided significance level of 0.05. No adjustments for multiplicity were considered in the present analyses.

## Results

### Participants

**Fig. 1:**
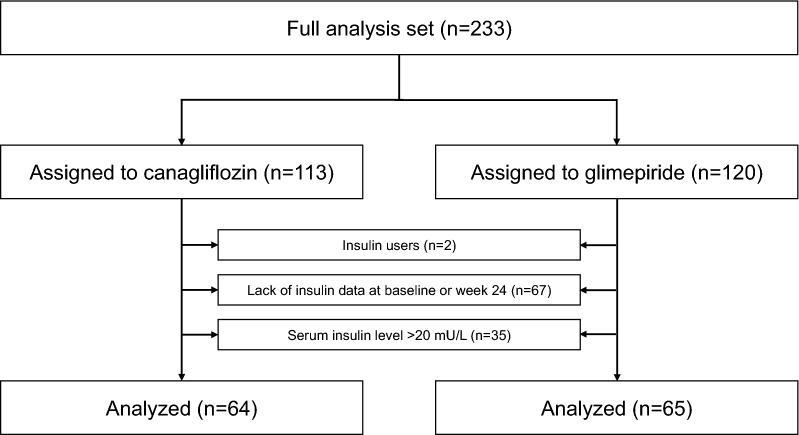
Flow chart of study participants

The flow chart of study participants is shown in Fig. 1. Among the FAS population (canagliflozin, n = 113; glimepiride, n = 120), two subjects were using insulin at baseline and were excluded from the analysis. In addition, 67 subjects were excluded due to lack of serum insulin data at baseline or week 24, and 35 subjects were excluded for serum insulin level > 20 mU/L. Finally, a total of 129 subjects (canagliflozin, n = 64; glimepiride, n = 65) were included in the present analysis. Baseline demographic and clinical characteristics of the analysis population are shown in Table 1. Regarding the HF-related parameters, the level of NT-proBNP was modest (median 228.0 [interquartile range 72.0− 421.0] pg/mL). Most patients had preserved LVEF and low/mild NYHA classes. Ischemia was the cause of HF in about half of the subjects. Regarding the T2D-related parameters, mean HbA1c was 6.8 ± 0.7%, and about half of the subjects had been receiving DPP-4 inhibitors, while about 40% of the subjects had not been taking any glucose-lowering agents at baseline.

**Table 1:** Baseline demographic and clinical characteristics of participants

Variable*	Overall (n = 129)	Canagliflozin (n = 64)	Glimepiride (n = 65)
Age, years	69.0 ± 9.4	68.7 ± 9.6	69.4 ± 9.3
Female, n (%)	32 (24.8)	16 (25.0)	16 (24.6)
BMI, kg/m^2^	24.9 ± 3.4	24.9 ± 3.3	24.9 ± 3.5
eGFR, mL/min/1.73m^2^	64.7 ± 14.3	64.6 ± 14.1	64.7 ± 14.7
NT-proBNP, pg/mL	228.0 (72.0− 421.0)	224.0 (72.0− 375.0)	239.0 (80.0− 455.0)
LVEF, %	59.0 ± 14.1	60.7 ± 12.4	57.3 ± 15.5
< 50%, n (%)	26 (20.2)	11 (17.2)	15 (23.1)
NYHA class, n (%)			
I	100 (77.5)	50 (78.1)	50 (76.9)
II	27 (20.9)	14 (21.9)	13 (20.0)
III	1 (0.8)	0 (0.0)	1 (1.5)
Unknown	1	0	1
Heart failure cause, n (%)			
Ischemia	66 (51.2)	37 (57.8)	29 (44.6)
Non-ischemia	63 (48.8)	27 (42.2)	36 (55.4)
Glucose, mg/dL	137.7 ± 31.5	135.3 ± 27.8	140.0 ± 34.8
HbA1c, %	6.8 ± 0.7	6.8 ± 0.7	6.9 ± 0.8
Medication for T2D, n (%)			
Metformin	23 (17.8)	8 (12.5)	15 (23.1)
DPP-4 inhibitor	62 (48.1)	33 (51.6)	29 (44.6)
Other	26 (20.2)	12 (18.8)	14 (21.5)
Insulin	0 (0.0)	0 (0.0)	0 (0.0)
Medication-naïve	53 (41.1)	26 (40.6)	27 (41.5)

*Data are mean ± standard deviation or median (interquartile range) unless otherwise noted

*BMI* body mass index, *DPP-4* dipeptidyl peptidase-4, *eGFR* estimated glomerular filtration rate, *LVEF* left ventricular ejection fraction, *NT-proBNP* N-terminal pro-brain natriuretic peptide, *NYHA* New York Heart Association, *T2D* type 2 diabetes

### Comparison of glycemic and insulin indices between groups

The mean changes from baseline to week 24 in HbA1c were 0.12% (95% confidence interval [**CI**], − 0.06 to 0.30) in the canagliflozin group and − 0.30% (95% CI, − 0.48 to –0.11) in the glimepiride group (between-group difference [canagliflozin minus glimepiride] 0.42% [95% CI, 0.16 to 0.68]; p = 0.002). The mean changes in glucose levels were –6.84 mg/dL (95% CI, − 14.03 to 0.34) in the canagliflozin group and − 13.08 mg/dL (95% CI, − 20.20 to − 5.95) in the glimepiride group (between-group difference 6.23 mg/dL [95% CI, − 3.89 to 16.35]; p = 0.227). These results were similar to those observed in the overall CANDLE trial population [22].

**Fig. 2:**
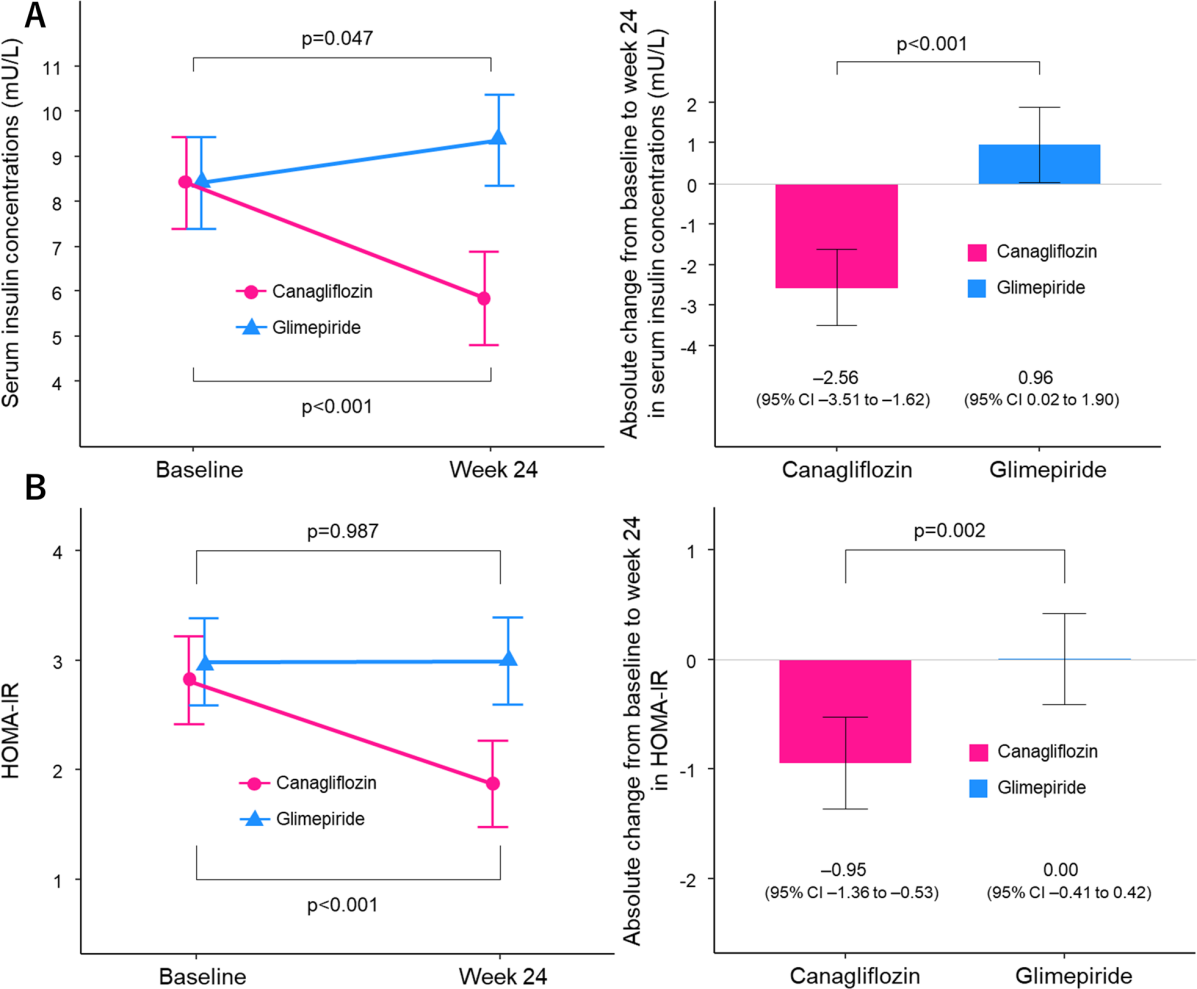
Changes from baseline to week 24 in serum insulin concentrations and HOMA-IR. A Serum insulin concentrations. B HOMA-IR. The data are expressed as the absolute change (mean and 95% confidence interval) from baseline to week 24. *HOMA-IR* homeostasis model assessment of insulin resistance

The mean insulin levels at baseline were 8.4 ± 3.9 mU/L in the canagliflozin group and 8.4 ± 4.7 mU/L in the glimepiride group. Serum insulin concentrations at week 24 were significantly reduced in the canagliflozin group and increased in the glimepiride group, with a between-group difference of − 3.52 mU/L (95% CI, − 4.85 to − 2.19; p < 0.001, Fig. 2A). Canagliflozin treatment also reduced HOMA-IR, while glimepiride treatment did not, with a between-group difference − 0.95 (95% CI, − 1.54 to − 0.36; p = 0.002, Fig. 2B).

### Association between changes in serum insulin concentrations and clinical parameters of interest

**Fig. 3:**
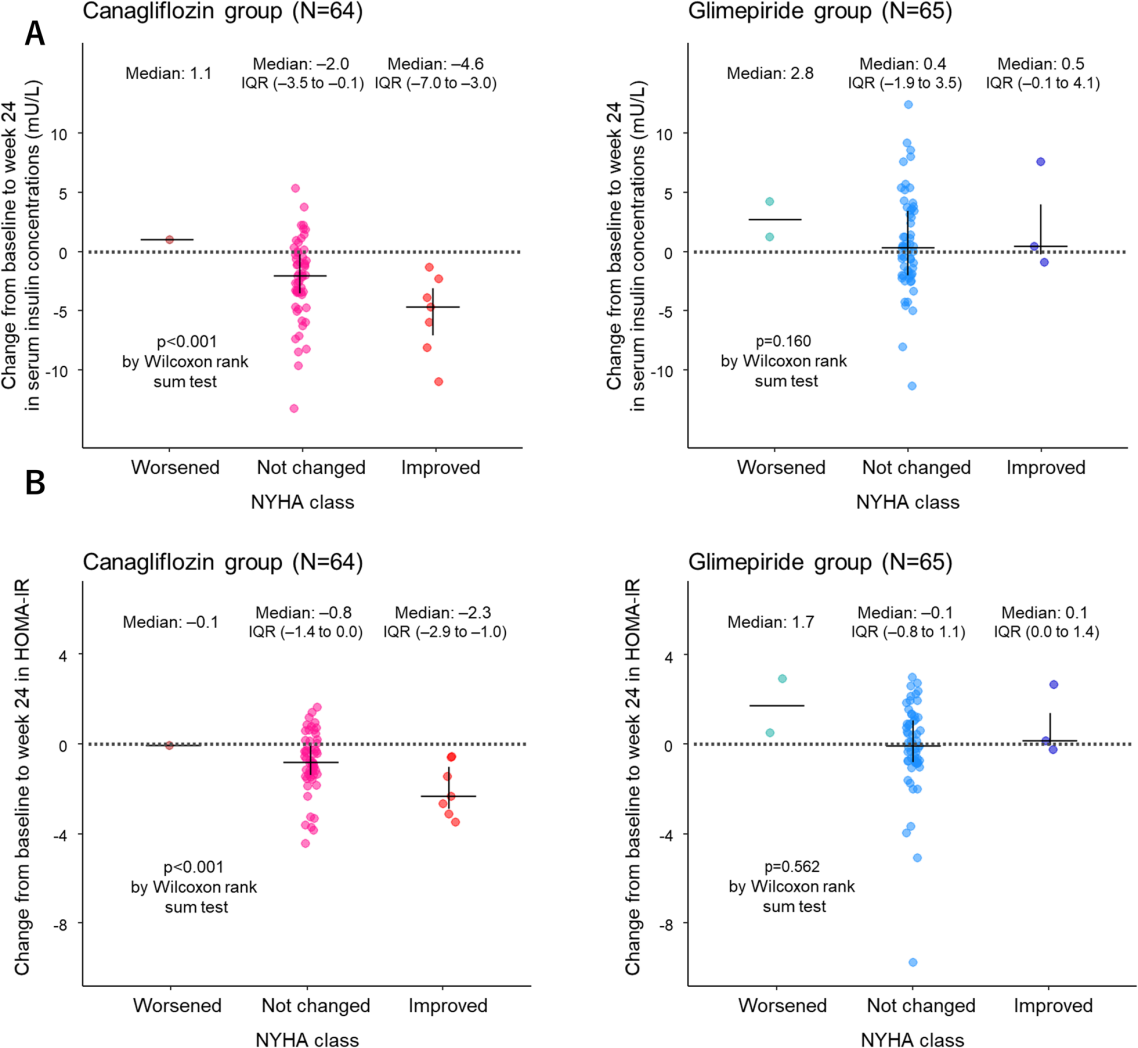
Associations between changes in insulin indices and NYHA class. The data are expressed as the median (interquartile range) change from baseline to week 24 in serum insulin concentrations **A** and HOMA-IR **B** in subgroups stratified by the categorical changes in NYHA class at week 24. *HOMA-IR* homeostasis model assessment of insulin resistance, *NYHA* New York Heart Association

Pearson’s correlations between changes in serum insulin concentrations and clinical parameters of interest from baseline to week 24 are shown in Table 2. In the canagliflozin group, changes in serum insulin concentrations were significantly correlated with those in systolic blood pressure (**SBP**), but not in other parameters, such as body mass index, lipid profiles, and NT-proBNP. Changes in serum insulin concentrations were also significantly associated with categorical changes in the NYHA class in the canagliflozin group, but not in the glimepiride group (Fig. 3A). This was also observed in the analyses to assess the association between changes in HOMA-IR and NYHA class (Fig. 3B).

**Table 2:** Pearson’s correlations between changes in serum insulin concentrations and clinical parameters of interest from baseline to week 24

Parameter	Canagliflozin (n = 64)		Glimepiride (n = 65)	
	Coefficient	p-value	Coefficient	p-value
SBP	0.335	0.007	− 0.001	0.995
BMI	0.102	0.425	0.053	0.678
ePV	0.053	0.679	− 0.141	0.261
eGFR	0.128	0.314	0.024	0.850
HbA1c	− 0.199	0.116	− 0.044	0.727
Uric acid	0.098	0.442	0.136	0.280
Triglycerides	0.036	0.778	0.138	0.273
HDL-C	− 0.245	0.051	− 0.079	0.532
LDL-C	− 0.135	0.287	− 0.170	0.177
NT-proBNP*	− 0.032	0.807	− 0.060	0.636

^*^ Log-transformed

ePV, estimated plasma volume; HDL-C, high-density lipoprotein cholesterol; LDL-C, low-density lipoprotein cholesterol; SBP, systolic blood pressure. Others, see Table 1

### Effects of baseline serum insulin concentration on clinical measures of interest

**Fig. 4:**
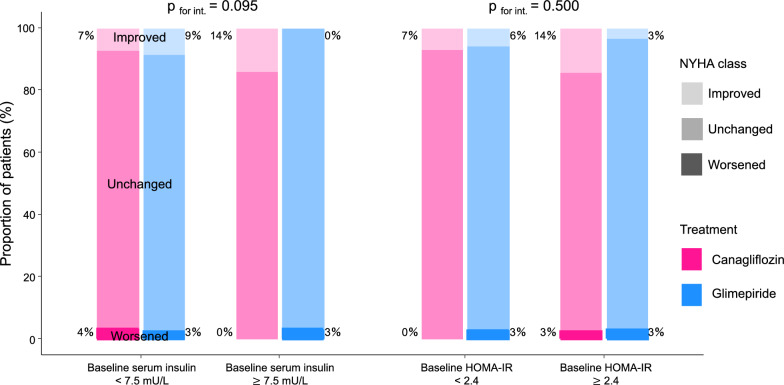
Changes from baseline in NYHA class at week 24 in subgroups stratified by baseline median serum insulin concentrations (left) and HOMA-IR (right). The numbers next to the bars indicate the frequency of cases in which NYHA improved or worsened at week 24. Between-subgroup differences in the treatment effect on NYHA class are analyzed as P_for interaction_. HOMA-IR, homeostasis model assessment of insulin resistance; NYHA, New York Heart Association

Between-group differences in changes from baseline to week 24 on clinical measures of interest in subgroups stratified by baseline serum insulin concentration (median 7.5 mU/L) are shown in Table 3. The treatment effects on those parameters did not differ between subgroups with baseline serum insulin concentration < 7.5 mU/L and ≥ 7.5 mU/L (all p for interaction > 0.09). The treatment effects on categorical changes in the NYHA class at week 24 were also similar according to the median serum insulin concentration at baseline (p for interaction = 0.095, Fig. 4, left). These findings were also similar when applying the HOMA-IR (Additional file 1 and Fig. 4, right).

**Table 3:** Between-group differences in changes at week 24 for clinical measures of interest in subgroups stratified by baseline serum insulin concentration

Parameter	Treatment effect measures	Baseline serum insulin concentration < 7.5 mU/L	Baseline serum insulin concentration ≥ 7.5 mU/L	p-value for interaction
SBP, mmHg	Difference (canagliflozin minus glimepiride) in change	3.082 (− 2.423 to 8.587)	− 1.621 (− 7.026 to 3.784)	0.232
BMI, kg/m^2^		− 0.933 (− 1.474 to − 0.393)	− 1.590 (− 2.130 to − 1.050)	0.092
ePV, %		− 6.500 (− 12.933 to − 0.066)	− 7.477 (− 13.847 to − 1.106)	0.832
eGFR, mL/min/1.73m^2^		0.679 (− 2.552 to 3.910)	− 1.736 (− 4.938 to 1.465)	0.298
HbA1c, %		0.229 (− 0.055 to 0.513)	0.498 (0.214 to 0.782)	0.189
Uric acid, mg/dL		− 1.057 (− 1.485 to − 0.655)	− 0.859 (− 1.266 to − 0.452)	0.498
Triglycerides, mg/dL		0.372 (− 29.247 to 29.990)	4.462 (− 25.061 to 33.985)	0.848
HDL-C, mg/dL		4.559 (1.366 to 7.752)	1.812 (− 1.352 to 4.976)	0.231
LDL-C, mg/dL		0.924 (− 7.180 to 9.029)	5.555 (− 2.498 to 13.608)	0.427
NT-proBNP*	Ratio (canagliflozin vs. glimepiride) of proportional change	0.946 (0.752 to 1.190)	0.977 (0.782 to 1.222)	0.840

*Log-transformed. Data are shown as mean (95% confidence interval)

Abbreviations, see Tables 1 and 2

## Discussion

Major findings in the present analysis of data from the randomized CANDLE trial in patients with T2D and CHF were as follows: (1) 24 weeks of treatment with canagliflozin, relative to glimepiride, significantly reduced serum insulin concentrations and HOMA-IR, (2) the decrease in insulin concentrations was significantly correlated with reductions in SBP (3) the decreases in insulin concentrations, and even HOMA-IR, were also significantly associated with improvement in the NYHA class in patients treated with canagliflozin, and (4) those effects of canagliflozin treatment on clinical measures did not differ according to baseline levels of insulin and HOMA-IR. These findings suggest that canagliflozin-induced attenuation of excess insulin overload explains, in part, the clinical benefits on HF-related outcomes observed in recent CVOTs with SGLT2 inhibitors.

Systemic insulin resistance also causes chronic activation of local insulin signaling and energy disturbances in cardiac tissues, resulting in the development and deterioration of HF [4–7]. Thus, insulin abnormalities and insulin resistance are key drivers of the development of HF in T2D, and thereby represent possible therapeutic targets [29–31]. However, there is little clinical evidence on whether therapeutic interventions for insulin resistance can improve HF-related status and outcomes [32]. Currently, two conventional glucose-lowering agents, metformin and thiazolidinedione, are well known to improve insulin resistance and cause cardiovascular benefits [33, 34]. Metformin treatment is associated with clinical benefits in patients with T2D and HF [35], although thiazolidinedione is not recommended in patients with or at risk of HF due to enhanced sodium reabsorption at the renal proximal tubule and a resultant increased risk of incident HF [36, 37]. However, the potential risk of HF in patients with T2D is still higher than that in a non-T2D population, which imposes an excess risk of morbidity and mortality [38, 39].

SGLT2 inhibitors are glucose-lowering agents that increase urinary glucose excretion [8]. This unique mode of action of SGLT2 inhibitors mitigates glucose toxicity independently of insulin secretion, thereby protecting pancreatic beta-cell function and relieving excess insulin overload. To date, several experimental and clinical studies have demonstrated an improvement in insulin resistance with SGLT2 inhibition [9–16]. Recent CVOTs with SGLT2 inhibitors demonstrated a consistent reduction in the risk of HF-related events in patients with T2D at high risk of CVD or HF irrespective of diabetes status [17–20]. These findings indicate that the therapeutic effects of SGLT2 inhibitors on HF-related outcomes are, at least in part, beyond glycemic control. Intriguingly, it is speculated that a modest increase of ketone body levels via SGLT2 inhibition plays beneficial roles in cardiac energetic alterations and amelioration of insulin resistance [21]. Thus, an improvement in insulin resistance accompanied by SGLT2 inhibitor treatment is likely to, at least in part, mediate the reduction in the risk of HF-related events.

In a recent substudy from the Empire HF trial for patients with left ventricular systolic dysfunction (LVEF ≤ 40%) with or without T2D [40], Jensen et al. for the first time revealed that 12 weeks of empagliflozin treatment, relative to placebo, improved both hepatic and peripheral insulin resistance, accompanied by significant reductions in body weight and lean mass. Results of the Empire HF trial previously demonstrated that empagliflozin also reduced estimated extracellular volume, ePV, and pulmonary capillary wedge pressure [26, 41], suggesting key mechanisms of SGLT2 inhibition underlying early and sustained clinical benefits for HF-related events. In the present study from the CANDLE trial, we also found that canagliflozin treatment alleviated hyperinsulinemia and insulin resistance in patients with T2D and HF almost exhibiting HF with preserved ejection fraction (HFpEF). Importantly, we also previously reported that canagliflozin treatment reduced ePV in the overall CANDLE trial population, and even in the HFpEF subpopulation [22]. These findings suggest that SGLT2 inhibitor treatment provides favorable hemodynamic and metabolic alterations in patients with HF, irrespective of LVEF category. This may support the robust clinical benefits seen in CVOTs for both HF phenotypes, systolic dysfunction and HFpEF, although profound pathophysiological mechanisms and molecular actions of SGLT2 inhibitors are likely to differ between phenotypes [31]. Given the multifaceted mechanisms potentially underlying such clinical benefits of SGLT2 inhibition [42, 43], however, whether improvement of hyperinsulinemia and insulin resistance via SGLT2 inhibition directly affects clinical manifestations and prognosis in patients with HF remains poorly understood.

Hyperinsulinemia up-regulates the expression of cardiac and renal sodium-hydrogen exchanger (**NHE**) isoforms, leading to cardiac dysfunction and renal sodium retention and excess body fluid burden [31, 44]. Recent experimental studies revealed that SGLT2 inhibition blocked NHE activation [45–47], and that is considered a promising mechanism underlying the clinical benefits of SGLT2 inhibitors for HF [31, 48]. Additionally, in our study, canagliflozin treatment alleviated hyperinsulinemia and insulin resistance, and those changes were associated with SBP reduction and improvement in NYHA class. This suggests that SGLT2 inhibitor treatment improved the clinical manifestations of HF via correction of hyperinsulinemia and insulin resistance located upstream of HF concomitant with T2D. To our knowledge, this is the first clinical report to show an SGLT2 inhibitor-mediated association between improvement in insulin resistance and relief of HF symptoms. However, our finding in this post-hoc analysis of the CANDLE trial may be still a hypothesis-generating. Further studies are needed to investigate whether the improvement in insulin homeostasis with SGLT2 inhibitor treatment has a direct impact on improving cardiac function and prognosis in patients with HF.

This present study has several potential limitations. First, this was a post-hoc analysis of the CANDLE trial that was not designed or powered to evaluate the effects of canagliflozin treatment on insulin-related parameters. Especially, the number of non-insulin users in the present analysis was small, although most participants (97%) to the entire CANDLE trial were non-insulin users. Second, although the study protocol specified fasting blood sampling, some insulin data were unlikely indicative of fasting sampling. To minimize the possibility of non-fasting blood sampling, the present analysis excluded subjects with insulin levels ≥ 20 mU/L, based on a previous report that fasting levels of plasma insulin were 11.2 ± 6.0 mU/L in Japanese patients with T2D [24]. Third, in general it would be quite reasonable that the HOMA-IR resultantly reduces after SGLT2 inhibitor treatment that decreases both serum insulin and glucose levels. It is currently uncertain whether HOMA-IR is a suitable method to assess insulin resistance in individuals receiving treatment with an SGLT2 inhibitor [49]. However, in the CANDLE trial, no data on other insulin resistance indices were assessed with gold-standard methods, such as the hyperinsulinemic-euglycemic clamp test, the Matsuda-DeFronzo index [50], and adipose insulin resistance index [51]. Further research would be therefore needed to assess the effects of SGLT2 inhibitor on those indices and their association with clinical HF status. Fourth, we have no detailed clinical information on events that would affect insulin levels, such as changes in glucose-lowering agents or addition of insulin treatment, during the follow-up period. Fifth, we were not able to evaluate an association of reductions in insulin levels and improvements in insulin resistance with the risk of HF-related events in the CANDLE trial. Finally, the CANDLE trial included Japanese patients with clinically stable T2D and HF, mostly of the HFpEF phenotype; therefore, generalizability of our findings to other clinical situations and populations is uncertain.

## Conclusion

Our findings suggest that in patients with T2D and HF, canagliflozin treatment ameliorated excess insulin overload and insulin resistance, contributing to the improvement in clinical HF status. This may explain, in part, the clinical benefits of SGLT2 inhibitors on HF-related outcomes.

## Supplementary Information

**Additional file 1.** Between-group differences in changes at week 24 for clinical measures of interest in subgroups stratified by baseline HOMA-IR.

## Data Availability

The datasets analyzed during the current study are available from the corresponding author upon reasonable request (tanakaa2@cc.saga-u.ac.jp).

## Abbreviations

CHF: Chronic heart failure
CI: Confidence interval
CVD: Cardiovascular disease
CVOT: Cardiovascular outcome trial
eGFR: Estimated glomerular filtration rate
ePV: Estimated plasma volume
FAS: Full analysis set
HF: Heart failure
HFpEF: Heart failure with preserved ejection fraction
HOMA-IR: Homeostasis model assessment of insulin resistance
LVEF: Left ventricular ejection fraction
NHE: Sodium-hydrogen exchanger
NT-proBNP: N-terminal pro-brain natriuretic peptide
NYHA: New York Heart Association
SBP: Systolic blood pressure
SGLT2: Sodium-glucose cotransporter 2
T2D: Type 2 diabetes
