# Supplementary material for: Association between serum insulin levels and heart failure-related parameters in patients with type 2 diabetes and heart failure treated with canagliflozin: a post-hoc analysis of the randomized CANDLE trial
Source: Cardiovasc Diabetol. 2022 Aug 8;21:151. doi: 10.1186/s12933-022-01589-3 (PMC9358857; doi:10.1186/s12933-022-01589-3)
Supplement: Supplementary file 1 — Additional file 1. Between-group differences in changes at week 24 for clinical measures of interest in subgroups stratified by baseline HOMA-IR. [file 12933_2022_1589_MOESM1_ESM.docx]

**Additional file 1. Between-group differences in changes at week 24 for clinical measures of interest in subgroups stratified by baseline HOMA-IR**

| **Parameter** | **Treatment effect** | **Baseline HOMA-IR <2.4** | **Baseline HOMA-IR ≥2.4** | **p-value for interaction** |
| --- | --- | --- | --- | --- |
| SBP, mmHg | Difference (canagliflozin minus glimepiride) in change | 3.153 (‒2.336 to 8.642) | ‒2.346 (‒7.784 to 3.091) | 0.163 |
| BMI, kg/m^2^ |  | ‒0.895 (‒1.432 to ‒0.357) | ‒1.622 (‒2.159 to ‒1.086) | 0.060 |
| ePV, % |  | ‒4.919 (‒11.328 to 1.490) | ‒8.552 (‒14.903 to ‒2.202) | 0.430 |
| eGFR, mL/min/1.73m^2^ |  | ‒0.025 (‒3.258 to 3.209) | ‒0.929 (‒4.136 to 2.277) | 0.697 |
| HbA1c, % |  | 0.247 (‒0.038 to 0.532) | 0.470 (0.191 to 0.749) | 0.273 |
| Uric acid, mg/dL |  | ‒1.071 (‒1.472 to ‒0.670) | ‒0.852 (‒1.254 to ‒0.451) | 0.451 |
| Triglycerides, mg/dL |  | 5.955 (‒23.299 to 35.208) | ‒3.834 (‒33.300 to 25.632) | 0.644 |
| HDL-C, mg/dL |  | 3.041 (‒0.169 to 6.251) | 3.123 (‒0.064 to 6.309) | 0.972 |
| LDL-C, mg/dL |  | ‒0.088 (‒8.162 to 7.986) | 6.944 (‒1.051 to 14.940) | 0.225 |
| NT-proBNP* | Ratio (canagliflozin vs. glimepiride) of proportional change | 0.937 (0.745 to 1.179) | 1.002 (0.800 to 1.254) | 0.684 |

* Log-transformed. Data are shown as mean (95% confidence interval).

BMI, body mass index; eGFR, estimated glomerular filtration rate; ePV, estimated plasma volume; HDL-C, high-density lipoprotein cholesterol; HOMA-IR, homeostasis model assessment of insulin resistance; LDL-C, low-density lipoprotein cholesterol; NT-proBNP, N-terminal pro-brain natriuretic peptide; SBP, systolic blood pressure.
